# Heart Failure Knowledge Assessment and Perceived Patient Satisfaction in Heart Failure Units: A Multicenter Observational Survey

**DOI:** 10.31083/j.rcm2509328

**Published:** 2024-09-13

**Authors:** Josebe Goirigolzarri-Artaza, Marta Cobo-Marcos, Laura Peña-Conde, Adolfo Villa, Diego Iglesias, Alberto Esteban-Férnandez, Fátima de la Torre, Jesús Álvarez-García, Aitor Hérnandez-Hernández, Juan Górriz-Magaña, Rocío Ayala, Mikel Taibo-Urquía, Cristina Beltrán, Pablo Díez-Villanueva, María Alejandra Restrepo-Córdoba, Julia González González, Ángel Manuel Iniesta Manjavacas, Sara Corredera-García, Sergio García-Gómez, María González-Piña, Álvaro Gamarra, Manuel Martínez-Sellés

**Affiliations:** ^1^Cardiology Department, Hospital Clínico San Carlos, 28040 Madrid, Spain; ^2^Cardiology Department, Hospital Universitario Puerta de Hierro, 28222 Madrid, Spain; ^3^Cardiology Department, Hospital Universitario La Paz, 28046 Madrid, Spain; ^4^Cardiology Department, Hospital Universitario Gregorio Marañón, 28007 Madrid, Spain; ^5^Cardiology Department, Hospital Universitario Sureste, 28500 Madrid, Spain; ^6^Cardiology Department, Hospital Universitario Infanta Sofía, 28702 Madrid, Spain; ^7^Cardiology Department, Hospital Universitario Severo Ochoa, 28911, Madrid, Spain; ^8^Cardiology Department, Hospital Universitario de Móstoles, 28935 Madrid, Spain; ^9^Cardiology Department, Hospital Universitario Ramón y Cajal, 28034 Madrid, Spain; ^10^CIBER CV, Centro de Investigación en Red en Enfermedades Cardiovasculares, 28029 Madrid, Spain; ^11^Cardiology Department, Clínica Universitaria de Navarra (sede Madrid), 28027 Madrid, Spain; ^12^Cardiology Department, Hospital Universitario Central de la Defensa Gómez Ulla, 28047 Madrid, Spain; ^13^Cardiology Department, Hospital de la Cruz Roja San José y Santa Adela, 28003 Madrid, Spain; ^14^Cardiology Department, Hospital Universitario Fundación Jiménez Díaz, 28040 Madrid, Spain; ^15^Cardiology Department, Hospital Universitario Infanta Leonor, 28031 Madrid, Spain; ^16^Cardiology Department, Hospital Universitario La Princesa, 28006 Madrid, Spain; ^17^Instituto de Investigación Sanitaria Gregorio Marañón, 28007 Madrid, Spain; ^18^Universidad Europea, 28005 Madrid, Spain; ^19^School of medicine, Complutense University, 28040 Madrid, Spain

**Keywords:** knowledge, perceived level of satisfaction, patient reported experience, heart failure, patient

## Abstract

**Background:**

Self-care and empowerment promotion in patients with heart failure (HF) is essential for improving their prognosis, but there is limited information concerning the patients' depth of knowledge about this pathology as well as patient satisfaction within heart failure units (HFUs). Our objective was to assess both aspects in a cohort of patients regularly followed-up HFUs.

**Methods:**

A multicenter, observational study was conducted with consecutive patients followed in 14 HFUs between June and November 2023. It was based on a cross-sectional survey comprising 23 questions related to demographics, knowledge/self-care, and the subjective assessment of perceived quality and satisfaction in HFUs.

**Results:**

281 patients were included (36.7% women, 74.7% aged over 65 years). 48% had hospitalizations for HF or sought emergency department services within the preceding year. The mean correct responses related to knowledge were 9.7 ± 2.3 (80.7% of the total), and 53 patients (18.9%) answered all knowledge questions correctly. 211 (79.6%) could identify potential HF decompensation with abrupt weight gain, and 196 (74.2%) recognized at least three additional signs of worsening HF. 266 patients (98.2%) were likely or very likely to recommend HFUs, and 194 (89.8%) positively appreciated the experience at the day hospital.

**Conclusions:**

Patients followed up in HFUs showed adequate but improvable knowledge and capacity for self-care, with a high level of satisfaction.

## 1. Introduction 

Heart failure (HF) is a complex clinical syndrome. Its prevalence is clearly increasing due to the progressive aging of the population, as well as the improved treatment of acute heart diseases that lead to chronic conditions such as HF. The follow-up and management of these patients constitutes a significant challenge, and current guidelines recommend follow-up in heart failure units (HFUs) with the aim of reducing hospital admissions as well as morbidity and mortality [1]. Within this follow-up, it is essential to promote patient knowledge and self-care. Self-care in HF refers to the actions that patients undertake to maintain their well-being and health, and to address potential complications (with the assistance of healthcare professionals) that may arise from this condition [2]. This includes medication adherence, physical exercise, symptom monitoring, and often daily weight monitoring, as well as dietary measures such as avoiding excessive fluid intake. Patients who are actively engaged in self-care and adherence exhibit better prognosis, with reduced mortality and fewer hospitalizations due to HF [3]. Numerous programs and various schemes have been developed to promote patient self-care, including remote monitoring, educational sessions, or home nursing visits. These programs have shown to improve prognosis in HF, although results among them are heterogeneous, and there is no single model in the literature that has proven to be superior to others as an educational tool [4]. As a result, most HFUs provide general recommendations for HF, and each center conducts a different educational intervention. Objective tools exist to measure the degree of self-care and knowledge in HF, but they are not routinely used. Consequently, it is common to overlook the actual level of disease knowledge among patients followed up in HFUs [5, 6, 7, 8].

On the other hand, HFUs allow for closer patient monitoring during medical consultations and managing worsening HF in day hospital (DH). However, the degree of satisfaction reported by patients in DH and the understanding of information received during medical visits in HFUs are also unknown. Therefore, the main objectives of our study were to evaluate the overall knowledge of HF among patients followed in different HFUs and to determine the perceived satisfaction level with care during medical visits and worsening HF management in the DH.

## 2. Materials and Methods

### 2.1 Study Design

This was an observational, multicenter study of HFUs corresponding to fourteen hospitals. A cross-sectional survey was conducted including consecutive patients between June and November 2023. Patients with a confirmed diagnosis of HF according to current clinical practice guidelines were included [1]. The exclusion criterion was the presence of moderate to severe dementia (established diagnosis) or any other condition that would hinder reliable responses to the survey questions. Patients (and/or caregivers as necessary) were administered a survey to complete, which was then collected by the research staff.

### 2.2 Survey Characteristics

The survey was conducted by a team of cardiologists specialized in HF and experienced in this field (MICADO group) [9, 10]. The survey consists of 23 simple and easily reproducible questions (**Supplementary Material 1**) related to patients’ demographic aspects (questions 1–5), knowledge about symptomatology and treatment, as well as adherence to prescribed recommendations (questions 6–17), and the perceived quality and satisfaction level in HFUs medical and DH visits (questions 18–23). Only patients who had experienced a worsening HF episode during follow-up and required attention at DH responded to question 22. To identify patients’ knowledge of HF, questions 6–17 regarding HF knowledge were categorized as correct/incorrect responses, and patients who answered all questions correctly were identified. Additionally, patients who had positive responses to questions 18–23 were considered to have a higher level of satisfaction and understanding.

Furthermore, the New York Heart Association (NYHA) functional class, the presence of HF admissions or worsening HF requiring intravenous diuretic treatment in the last 12 months, and HFU follow-up time were collected. Additionally, patients’ educational level, defined as basic (primary and secondary school), middle high (high school and vocational training) and high (university) was collected. Finally, variables related to the characteristics of each center and HFUs included in the study were collected. The Consensus-Based Checklist for Reporting of Survey Studies (CROSS) checklist and the STROBE checklist were used to communicate the results [11, 12].

### 2.3 Statistical Analysis

This is a descriptive study in which categorical variables are expressed as numbers and percentages, and quantitative variables are presented as mean and standard deviation. Student’s *t*-test was used for the comparison of quantitative variables, and the chi-square test or Fisher’s exact test was employed for categorical variables. The STATA software (version 14, Stata Corp, College Station, TX, USA) was utilized for the analysis. The sample size calculation was based on the outcome obtained from the study conducted by Vidán MT, with a prevalence of moderate to high disease knowledge of 77.4%. Under this assumption, and for a confidence level of 95% and a precision of 5%, a sample size of 269 surveys was required [13].

## 3. Results

### 3.1 Clinical and Demographic Variables

A total of 281 patients completed the survey. Patients responded to almost all the questions (<5% blank responses in each question) except for question number 22, in which 65 patients (23.1%) did not respond. The main clinical and demographic characteristics of the patients are shown in Table 1. 42.3% were aged between 65 and 79 years, 37% were women, and nearly half of the patients (48.5%) had a basic educational level. 135 patients (48%) experienced HF admission or visit to the DH in HFUs requiring intravenous diuretics in the previous year, and 156 (55.5%) had follow-up exceeding one year.

**Table 1.** **Clinical and demographic characteristics**.

Variable		Frequency (%)
Age (years)		
	<65	71 (25.3%)
	65–79	119 (42.3%)
	>80	91 (32.4%)
Sex		
	Male	178 (63.3%)
	Female	103 (36.7%)
Educational level		
	Basic	133 (48.5%)
	Middle-High	68 (24.8%)
	High	73 (26.7%)
Living		
	Alone	56 (19.9%)
	With family	225 (80.1%)
Autonomous medication management		
	Yes	225 (80.6%)
	No (caregiver)	54 (19.4%)
NYHA		
	I	63 (22.9%)
	II	167 (60.7%)
	III	44 (16.0%)
	IV	1 (0.4%)
HF admission or iv diuretics at DH in HFU (12 months)		
	Yes	135 (48.0%)
	No	146 (52.0%)
Length of HFU follow-up		
	<6 months	86 (30.6%)
	6–12 months	39 (13.9%)
	>12 months	156 (55.5%)

DH, day hospital; HF, heart failure; HFU, heart failure unit; iv, intravenous; NYHA, New York Heart Association.

### 3.2 HFU and Center Characteristics

An average of 20.7 ± 2.46 surveys per center were included. Table 2 presents specific variables for each center. Thirteen out of fourteen centers had specialized HF nursing, and all centers conducted a reassessment of HF knowledge in patients. However, no center performed a specific, objective, and standardized evaluation of HF knowledge.

**Table 2.** **Specific characteristics of the heart failure centers and units included**.

Health care area (inhabitants)		
	<100,000	37 (13.2%)
	100,000–300,000	99 (35.2%)
	300,000–500,000	74 (26.3%)
	>500,000	71 (25.3%)
Type of hospital		
	Primary	17 (6.1%)
	Secondary	95 (33.8%)
	Tertiary	169 (60.1%)
Unit		
	Cardiology department	143 (50.9%)
	Multidisciplinary	138 (49.1%)
Unit (complexity)		
	Community	41 (14.6%)
	Specialized	193 (68.7%)
	Advanced	47 (16.7%)

### 3.3 Survey Results

#### 3.3.1 HF Knowledge and Self-Care

Heart failure knowledge and self-care responses are shown in Table 3. The mean correct responses related to knowledge were 9.7 ± 2.3 out of 12 (80.7% of the total), and 53 patients (18.9%) answered all questions correctly (questions 6–17). 63.2% of patients weighed themselves daily, and 80.1% knew the goal of daily weight measurement, increasing to 89.7% among patients who weighed themselves daily. When faced with sudden weight gain or the appearance of edema in lower limbs, 79.3% indicated they would increase furosemide. When considering only patients who identified weight gain as potential HF decompensation, this percentage increased to 91.7%, and 87% would also increase it in the case of edema in the lower limbs.

**Table 3.** **Heart failure knowledge and self-care responses**.

Questions	Response
Regarding physical exercise…	5.8% It might worsen my fatigue and heart failure
	94.2% Yes, I should practice exercise within my possibilities
Do you weigh yourself every day?	63.2% Yes
	11.4% No
	25.4% Occasionally
When should you weigh yourself?	91% Right after waking up
	9% Anytime during the day
Do you know why you weigh yourself?	80.1% Yes, it’s useful to see if I’m retaining fluid
	12% Yes, it helps to avoid gaining weight and adjust food intake
	7.9% No
A very rapid weight gain may indicate	9.8% I have eaten excessively
	79.6% I’m retaining fluids
	10.6% I don’t know
In the previous case, would you?	20.7% Eat less
	79.3% Increase the prescribed furosemide
If your legs swell over a few days	20.8% Elevate my legs
	79.2% Increase the prescribed diuretic
Which is a warning sign of worsening heart failure	15.9% Swollen legs
	7.2% Shortness of breath in bed
	2.7% Rapid decrease in the amount of urine
	74.2% All of the above
Do you know what furosemide is for?	2.2% Yes, it increases heart strength
	89.3% Yes, it increases urine output
	8.5% No
Do you know what the anticoagulant medication is for?	77.5% Yes, it prevents clots from forming and traveling to different parts of the body
	6.7% Yes, it improves heart strength
	15.8% No, I’m not sure
Do you know what sacubitril/valsartan or ACE inhibitors, or ARBs are for?	7.3% Yes, they prevent clots
	61.4% Yes, they relieve my heart’s workload and help it function better
	31.3% No, I don’t know
If you are prescribed pain medication…	12% There’s no problem with any medication prescribed
	88% I should avoid non-steroidal anti-inflammatories

ACE inhibitors, angiotensin-converting enzyme inhibitors; ARBs, angiotensin ii receptor blockers.

The ability of patients to answer all survey questions correctly was positively and significantly associated with NYHA functional class (32.6% all correct responses in NYHA III–IV compared to 17.6% in NYHA I–II, *p* = 0.03) and educational level (34.3% all correct responses in the higher educational group compared to 14.5% in the basic and medium group, *p* = 0.02). Additionally, those who answered all knowledge questions correctly showed a higher level of satisfaction (25.9% vs 8%, *p* < 0.01) and a lower percentage of admissions in the last 12 months (14.8% vs 25%, *p* = 0.04). However, there was no significant association between answering knowledge questions correctly and follow-up time in the HFU (23.3% all correct responses in follow-up less than a year and 16.9% in follow-up more than a year, *p* = 0.2).

#### 3.3.2 Satisfaction and Comprehension 

**Fig. 1.**
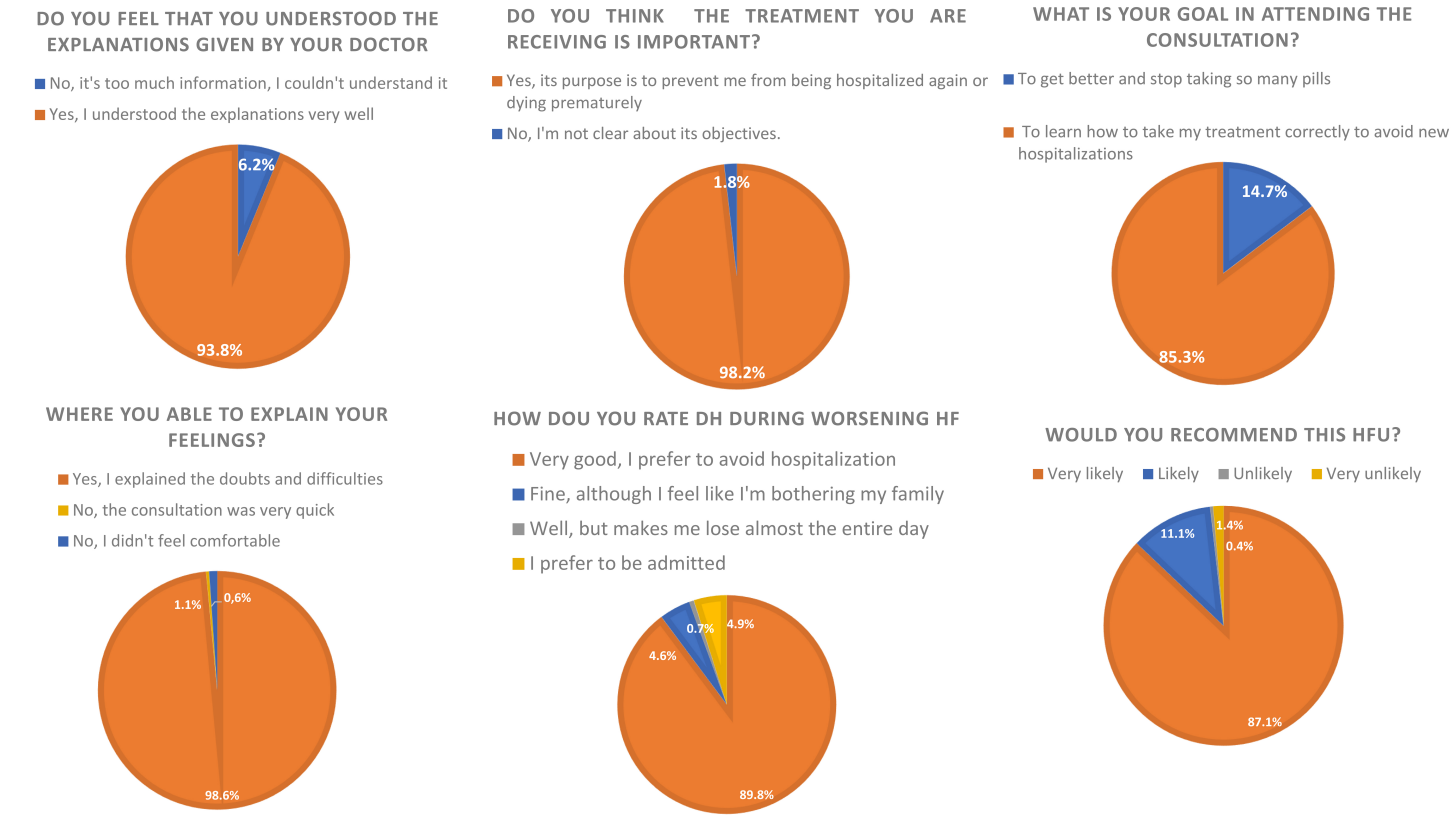
**Survey satisfaction and comprehension results (responses 18–23)**. The figure shows the level of comprehension and satisfaction perceived in HFUs as well as DH during worsening HF episodes. DH, day hospital; HF, heart failure; HFU, heart failure unit.

The degree of understanding of the information provided, and patient satisfaction were high (Fig. 1). 98.2% of surveyed patients would likely or very likely recommend the corresponding HFU. Only a significant association was found between a higher level of satisfaction and a higher educational level, with patients with a higher educational level showing a higher degree of satisfaction (82.8% higher level vs 61.7% basic and medium level, respectively, *p* = 0.001). Additionally, most patients (89.8%) positively perceived the care received at DH during worsening HF episodes.

#### 3.3.3 Other Results 

Patients in NYHA III–IV were older (25.8% were over 80 years old, 15.5% were between 65 and 79 years old, and 5.7% were under 65 years old, *p* < 0.05) and more frequently women (24.8% and 11.5%, respectively, *p* < 0.01), although there were no significant differences in educational level or autonomous medication management (*p* = 0.7 and *p* = 0.8, respectively). Patients who managed medication autonomously were younger, although there were no differences in knowledge responses (20.8% vs 19.5% correct responses, *p* = 0.8) or educational level (80.5% and 83.3% autonomous patients in basic vs medium/superior and superior education, respectively, *p* = 0.5).

## 4. Discussion

This study highlights the level of knowledge, self-care, and satisfaction in a cohort of patients followed in different HFUs, being the first multicenter Spanish study attempting to elucidate these crucial aspects in the follow-up of patients in HFUs.

Knowledge of HF was high, especially in specific aspects such as the importance of physical exercise or the contraindication of non-steroidal anti-inflammatory drugs. However, one in five surveyed patients did not know the reason for daily weight measurement, nor the significance of rapid weight gain or the appearance of edema in the lower limbs. A similar percentage also did not know what measures to take in response.

Patient knowledge in HF is crucial, as it is essential for proper self-care, and poor knowledge contributes to poorer self-care [14]. However, knowledge alone is insufficient for proper self-care, and prior education in HF only modestly modifies this capacity [15, 16, 17]. The results of our study support this concept. In our cohort, although 80.1% *knew* the purpose of daily weight measurement, only 63.2% *performed* it daily. Conversely, patients who weighed themselves daily more often knew this objective, more frequently identified decompensation, and were more likely to use a flexible diuretic regimen to reverse it. However, despite this, our study identified that there was still 8% who would not take any action despite identifying decompensation. Therefore, self-care is more complex than isolated education and includes social, psychological, and cultural aspects [17]. Furthermore, a meta-analysis on the efficacy of intervention programs aimed at improving self-care in patients with HF found that the longer duration of these programs (regardless of type) was the greatest determinant for improving self-care [4]. In our study, however, no differences were observed between the follow-up time and the number of correct responses.

Self-care and patient knowledge are not routinely measured objectively in HFUs. Several scales exist in the literature, with the European Heart Failure Self-Care Behaviour Scale (EHFScBS) and the Self-Care of Heart Fialure Index 7.2 (SCHFI) being the most commonly used and validated scales in studies [5, 6, 7, 8, 18]. However, even these scales have limitations, and the true ability to perform necessary self-care measures is poor. In the study by Vidán *et al*. [13], the correlation between responses obtained in the European Self-care Behaviour Scale and the actual ability to identify and carry out various measures was low. A recently published study has demonstrated that a HF knowledge survey allows quantifying and improving learning compared to the usual method, enhances self-care, and appears to confer some prognostic improvement [7]. In these scales, educational level poses a certain limitation and may play a role in the results and applicability of these scales. In fact, in our study, patients with a higher educational level responded better to HF knowledge questions. However, educational interventions and HF programs have shown to improve self-care across all educational levels and even across various socioeconomic strata, highlighting the importance of extending and customizing education to enhance health outcomes even further [16, 17, 19].

The scales mentioned above, such as the SCHFI, are validated scales that assess some of the variables included in this survey, such as daily weight or extra diuretic intake. However, aspects such as the degree of real HF knowledge as well as satisfaction and understanding in follow-up care in HFUs are not completely analyzed in these scales. Our survey expands its focus to these issues, which have been scantily investigated directly in the literature [5, 6, 7, 8, 18]. In HF, patient-centered experience is increasingly important, and our aim was to understand what patients felt and what motivated them. 93.8% felt they understood the instructions received adequately, and 98.6% were able to explain their doubts and difficulties to the doctor, with 98.2% of surveyed patients likely or very likely to recommend the corresponding HFU. Patients who answered all questions correctly had a higher degree of satisfaction, and this was positively associated with a higher level of education. Finally, another novel aspect is the assessment of DH from the patient’s experience (only patients with worsening HF episodes during follow-up answered). This allows avoiding HF admissions at the expense of frequent visits, which can be tiring or difficult in terms of family conciliation, especially in elderly patients who frequently attend with family members or caregivers. Most patients (89.8%) rated it positively and preferred this option to admission, which was the feeling perceived by only 4.8%.

The main limitation of our study was its cross-sectional nature, which does not allow us to see the implication of such a survey on HF outcomes. Additionally, we cannot rule out that patients with a low educational level may have refused to participate in the study due to the difficulty of understanding the survey, in the same way that non-adherent patients may have declined its participation. Besides, we did not employ validated self-care scales, which may limit the generalization of our results. However, we decided to conduct this survey to concurrently analyze additional aspects that were not comprehensively addressed in these surveys, such as the degree of satisfaction in HFUs follow-up. Finally, other patient characteristics with prognostic impact in HF, such as frailty or other geriatric syndromes, which may be related to patients’ ability to respond correctly, were not included [20].

## 5. Conclusions

In conclusion, patients followed up in the included HFUs exhibited adequate but improvable knowledge and self-care capabilities, along with a high level of understanding and satisfaction.

## Availability of Data and Materials

All data and materials are available on the submitted article.

## Abbreviations

DH, day hospital; HF, heart failure; HFU, heart failure unit; NYHA, New York Heart Association.

## Supplementary Material

Supplementary material associated with this article can be found, in the online version, at https://doi.org/10.31083/j.rcm2509328.

## References

[1] McDonagh TA, Metra M, Adamo M, Gardner RS, Baumbach A, Böhm M (2021). 2021 ESC Guidelines for the diagnosis and treatment of acute and chronic heart failure. *European Heart Journal*.

[2] Harkness K, Spaling MA, Currie K, Strachan PH, Clark AM (2015). A systematic review of patient heart failure self-care strategies. *The Journal of Cardiovascular Nursing*.

[3] van der Wal MHL, van Veldhuisen DJ, Veeger NJGM, Rutten FH, Jaarsma T (2010). Compliance with non-pharmacological recommendations and outcome in heart failure patients. *European Heart Journal*.

[4] Jonkman NH, Westland H, Groenwold RHH, Ågren S, Atienza F, Blue L (2016). Do Self-Management Interventions Work in Patients With Heart Failure? An Individual Patient Data Meta-Analysis. *Circulation*.

[5] Jaarsma T, Strömberg A, Mårtensson J, Dracup K (2003). Development and testing of the European Heart Failure Self-Care Behaviour Scale. *European Journal of Heart Failure*.

[6] van der Wal MHL, Jaarsma T, Moser DK, van Veldhuisen DJ (2005). Development and testing of the Dutch Heart Failure Knowledge Scale. *European Journal of Cardiovascular Nursing*.

[7] Sánchez-Ramos JG, Lerma-Barba MD, Segura-Rodríguez D, Pardo-Cabello A, Molina-Ruiz MT, Burillo-Gómez F (2022). Evaluación de un cuestionario de conocimientos en insuficiencia cardíaca y su utilidad para guiar la intervención educativa. *Revista Clínica Española*.

[8] Lainscak M, Keber I (2005). Validation of self assessment patient knowledge questionnaire for heart failure patients. *European Journal of Cardiovascular Nursing*.

[9] Álvarez-García J, Cristo-Ropero MJ, Iniesta-Manjavacas ÁM, Díez-Villanueva P, Esteban-Fernández A, De Juan Bagudá J (2023). Opinión sobre la guía ESC 2021 sobre insuficiencia cardiaca. Una encuesta a 387 médicos. *REC CardioClinics*.

[10] Álvarez-García J, Cristo Ropero MJ, Iniesta Manjavacas ÁM, Díez-Villanueva P, Esteban-Fernández A, de Juan Bagudá J (2023). Do Women Physicians Accept and Follow Heart Failure Guidelines More Than Men. *Current Heart Failure Reports*.

[11] Sharma A, Minh Duc NT, Luu Lam Thang T, Nam NH, Ng SJ, Abbas KS (2021). A Consensus-Based Checklist for Reporting of Survey Studies (CROSS). *Journal of General Internal Medicine*.

[12] von Elm E, Altman DG, Egger M, Pocock SJ, Gøtzsche PC, Vandenbroucke JP (2008). The Strengthening the Reporting of Observational Studies in Epidemiology (STROBE) statement: guidelines for reporting observational studies. *Journal of Clinical Epidemiology*.

[13] Vidán MT, Martín Sánchez FJ, Sánchez E, Ortiz FJ, Serra-Rexach JA, Martínez-Sellés M (2019). Most elderly patients hospitalized for heart failure lack the abilities needed to perform the tasks required for self-care: impact on outcomes. *European Journal of Heart Failure*.

[14] Powell LH, Calvin JE, Richardson D, Janssen I, Mendes de Leon CF, Flynn KJ (2010). Self-management counseling in patients with heart failure: the heart failure adherence and retention randomized behavioral trial. *JAMA*.

[15] Clark AM, Freydberg CN, McAlister FA, Tsuyuki RT, Armstrong PW, Strain LA (2009). Patient and informal caregivers’ knowledge of heart failure: necessary but insufficient for effective self-care. *European Journal of Heart Failure*.

[16] Jaarsma T, Strömberg A (2019). We told you so: ‘knowledge is not enough to improve heart failure self-care behaviour’. *European Journal of Heart Failure*.

[17] González B, Lupón J, Domingo MDM, Cano L, Cabanes R, de Antonio M (2014). Educational level and self-care behaviour in patients with heart failure before and after nurse educational intervention. *European Journal of Cardiovascular Nursing*.

[18] Vellone E, De Maria M, Iovino P, Barbaranelli C, Zeffiro V, Pucciarelli G (2020). The Self-Care of Heart Failure Index version 7.2: Further psychometric testing. *Research in Nursing & Health*.

[19] Capdevila Aguilera C, Vela Vallespín E, Clèries Escayola M, Yun Viladomat S, Fernández Solana C, Alcober Morte L (2023). Population-based evaluation of the impact of socioeconomic status on clinical outcomes in patients with heart failure in integrated care settings. *Revista Espanola De Cardiologia*.

[20] Jiménez-Méndez C, Díez-Villanueva P, Bonanad C, Ortiz-Cortés C, Barge-Caballero E, Goirigolzarri J (2022). Frailty and prognosis of older patients with chronic heart failure. *Revista Espanola De Cardiologia*.

