# Supplementary material for: Heart Failure Knowledge Assessment and Perceived Patient Satisfaction in Heart Failure Units: A Multicenter Observational Survey
Source: Rev Cardiovasc Med. 2024 Sep 13;25(9):328. doi: 10.31083/j.rcm2509328 (PMC11440440; doi:10.31083/j.rcm2509328)
Supplement: Supplementary file 1 [file 2153-8174-25-9-328-s1.zip › Supplementary Material 1.docx]

**SURVEY**

1. Age:
   1. Under 65 years old
   2. 65-79 years old
   3. 80 years old or older
2. Gender:
   1. Male
   2. Female
3. Educational level:
   1. Basic (Primary and secondary school)
   2. Middle-high (High school and vocational training)
   3. Higher (University)
4. Who do you live with?
   1. Alone
   2. With someone
5. Regarding medication management:
   1. I'm independent (I prepare and take my medication when needed)
   2. I'm not independent. Someone prepares and/or gives me the medication.
6. Regarding physical exercise, do you think:
   1. It's not good to exercise as it might worsen fatigue and my condition.
   2. I should exercise regularly within my capabilities.
7. Do you weigh yourself every day?
   1. Yes
   2. No
   3. Occasionally
8. When should you weigh yourself?
   1. Right after waking up, fasting, and always under the same conditions.
   2. Anytime during the day and with any amount of clothing.
9. Do you know why you have been advised (if applicable) to weigh yourself every day?
   1. Yes, it's useful to see if I'm retaining fluid.
   2. Yes, it helps to avoid gaining weight and adjust food intake if I'm gaining weight.
   3. No.
10. A very rapid weight gain (for example, 2 kg in two days or 4-5 kg in a week) may indicate that:
    1. I have eaten excessively during that week, so I should eat less.
    2. I'm retaining fluids due to my heart failure.
    3. I don't know what could be happening.
11. In the previous scenario, what would you do?
    1. Eat less to lose the gained weight.
    2. Increase the prescribed furosemide for a few days if recommended.
12. If your legs swell over a few days, what would you do?
    1. Elevate my legs and lie down to see if circulation improves and resolves.
    2. Increase the prescribed diuretic if recommended since I'm retaining fluids.
13. Which of the following symptoms is a warning sign?
    1. Swollen legs.
    2. Shortness of breath in bed.
    3. Rapid decrease in the amount of urine.
    4. All of the above.
14. Do you know what furosemide is for?
    1. Yes, it increases heart strength.
    2. Yes, it increases urine output (it's a diuretic).
    3. No.
15. Do you know what the anticoagulant medication (if you are using it) such as warfarin, dabigatran, edoxaban, rivaroxaban, apixaban is for?
    1. Yes, it prevents clots from forming and traveling to different parts of the body.
    2. Yes, it improves heart strength.
    3. No, I'm not sure.
16. Do you know what sacubitril/valsartan or ACE inhibitors or ARBs are for?
    1. Yes, they prevent clots.
    2. Yes, they relieve my heart's workload and help it function better.
    3. No, I don't know.
17. If you are prescribed pain medication when you have heart failure, what would you do?
    1. There's no problem with any medication prescribed. The important thing is to quickly relieve the pain.
    2. I should avoid non-steroidal anti-inflammatories like ibuprofen or dexketoprofen. I can take paracetamol or metamizole.
18. After the consultation, do you feel that you understood the explanations given by your doctor?
    1. No, it's too much information, and I couldn't understand it.
    2. Yes, I understood the explanations very well and how to take the treatment.
19. Do you think the pharmacological treatment you are receiving is important?
    1. Yes, it's important because its purpose is to prevent me from being hospitalized again or dying prematurely.
    2. No, I must take this treatment, but I'm not clear about its objectives.
20. What is your goal in attending the consultation?
    1. To get better so that I can stop taking so many pills as soon as possible.
    2. To learn how to take my treatment correctly and the necessary recommendations to avoid new hospitalizations or premature death.
21. After the consultation, do you feel that you were able to explain your feelings and concerns to the doctor?
    1. Yes, I explained to the doctor the doubts I had and the difficulties in carrying out what is good for me (for example, losing weight, weighing myself daily...)
    2. No, the consultation was very quick, and I didn't have time to ask anything.
    3. No, I didn't feel comfortable, I felt embarrassed to express my feelings.
22. If you have ever had fluid retention (heart failure decompensation) treated in the Day Hospital of the Heart Failure Unit, how do you rate it?
    1. Very good, I prefer to avoid hospitalization and be able to go home at the end of the morning after treatment.
    2. Fine, although it's tedious to come to the hospital for several days, and I feel like I'm bothering my family when they accompany me.
    3. Well, but going by ambulance several days in a row makes me lose almost the entire day, and I feel low-spirited.
    4. The fact of going for several consecutive days and spending several hours in the hospital sometimes makes me prefer to be admitted, as it is tiring and bothersome to my family/caregiver.
23. Would you recommend this unit to a friend or family member needing similar treatment?
    1. Very likely
    2. Likely
    3. Unlikely
    4. Very unlikely
